# Novel Analytical Method for Mix Design and Performance Prediction of High Calcium Fly Ash Geopolymer Concrete

**DOI:** 10.3390/polym13060900

**Published:** 2021-03-15

**Authors:** Chamila Gunasekara, Peter Atzarakis, Weena Lokuge, David W. Law, Sujeeva Setunge

**Affiliations:** 1 School of Engineering, Royal Melbourne Institute of Technology (RMIT) University, Melbourne, VIC 3000, Australia; 3166706@student.rmit.edu.au (P.A.); david.law@rmit.edu.au (D.W.L.); sujeeva.setunge@rmit.edu.au (S.S.); 2 School of Civil Engineering and Surveying, University of Southern Queensland, Springfield, QSL 4300, Australia; Weena.Lokuge@usq.edu.au

**Keywords:** high calcium fly ash, geopolymer concrete, artificial neural network, mix design, compressive strength, regression analysis

## Abstract

Despite extensive in-depth research into high calcium fly ash geopolymer concretes and a number of proposed methods to calculate the mix proportions, no universally applicable method to determine the mix proportions has been developed. This paper uses an artificial neural network (ANN) machine learning toolbox in a MATLAB programming environment together with a Bayesian regularization algorithm, the Levenberg-Marquardt algorithm and a scaled conjugate gradient algorithm to attain a specified target compressive strength at 28 days. The relationship between the four key parameters, namely water/solid ratio, alkaline activator/binder ratio, Na_2_SiO_3_/NaOH ratio and NaOH molarity, and the compressive strength of geopolymer concrete is determined. The geopolymer concrete mix proportions based on the ANN algorithm model and contour plots developed were experimentally validated. Thus, the proposed method can be used to determine mix designs for high calcium fly ash geopolymer concrete in the range 25–45 MPa at 28 days. In addition, the design equations developed using the statistical regression model provide an insight to predict tensile strength and elastic modulus for a given compressive strength.

## 1. Introduction

Concrete is the most widely utilised construction material in the world. It is essential in the urbanisation of society in order to improve human living standards [1]. The expansion of urbanization and the worldwide population increase has led to a significant enhancement of the current global cement production of 12% in 2019, which is predicted to double by 2050 [2]. China is dominating the global cement market and produced 2.4 billion tonnes in 2018, which accounted for half of the global cement demand, followed by India at 290 million tonnes [3]. The manufacture of one ton of cement can generate 0.6 to 1.0 ton of CO_2_ depending on the manufacturing method employed [4,5,6], and is responsible for the 5−9% of global CO_2_ emission [7,8,9,10].

Many researchers have been exploring alternative sustainable cementitious binders that can reduce the dependence on Portland cement (PC) in construction [11,12,13]. Fly ash geopolymer concrete is a promising alternative that can reduce CO_2_ emissions by 25‒45% by utilizing waste coal combustion products [14]. High calcium fly ash is a popular material for the production of alkali-activated concrete due to worldwide availability and containing sufficient quantities of reactive aluminate, silicate and calcium oxide [15,16]. European countries, such as Greece, Poland, and Spain generate the majority of the high calcium fly ash, derived from lignite coal production [17]. Greece produces 12 million tonnes of high calcium fly ash annually while in Asia (Thailand) generates about 3 million tonnes [16]. However, more than 60% of this fly ash is being discarded in landfill, posing serious environmental concerns [18].

Nuaklong et al. [19] investigated the compressive strength and fire-resistance of high calcium fly ash alkali-activated concrete blended with rice husk ash. The results showed that the 28-day compressive strengths of geopolymer ranged from 36.0 to 38.1 MPa due to an improved microstructure and denser matrix. However, the inclusion of SiO_2_ rich rice husk ash had an adverse effect on the postfire residual strength. Wongsa et al. [20] examined the fire resistance behaviours of high calcium fly ash alkali-activated concrete incorporating natural zeolite and mullite. Test results showed that the use of these additives alone and together improved the fire resistance of concrete, which was attributed to the presence of Ye’elimite and Wallastonite formed at high temperatures. Wong et al. [21] illustrated that high calcium fly ash-brick powder alkali-activated composite can yield up to 44.2 MPa at 28 days. However, the results indicated that brick powder replacement beyond 10% resulted in the creation of an inhomogeneous microstructure in concrete.

Research has shown that a range of mix design parameters influence the compressive strength of fly ash geopolymer concrete. Ling et al. [22] studied the impact of four design parameters, namely the SiO_2_/Na_2_O ratio, the alkali activator concentration, the liquid/fly ash ratio and curing temperature, on the setting time and compressive strength development of high calcium fly ash geopolymer. Test results confirmed that as the SiO_2_/Na_2_O ratio increased, the setting time was accelerated but the compressive strength was reduced. As activator concentration increased, the setting time for geopolymer mixes with SiO_2_/Na_2_O of 1.0 and 1.5 were prolonged but were shortened when SiO_2_/Na_2_O equalled 2.0, while the compressive strength of these geopolymer mixes increased. The data also showed that an elevated curing temperature increased the compressive strength. Zhang and Feng [23] reported that water content, NaOH molarity and curing temperature influenced the compressive strength development of high calcium fly ash geopolymers. Abdullah et al. [24] noted that NaOH molarity, Na_2_SiO_3_/NaOH ratio, fly ash/alkaline activator ratio and curing temperature affected the compressive strength of fly ash geopolymers. The test results revealed that a 12 M NaOH solution and mass ratios of fly ash/alkaline activator and Na_2_SiO_3_/NaOH of 2.0 and 2.5, respectively, yielded the highest compressive strength. Literature [25,26] has reported that a fly ash/alkaline activator ratio of 3.3−4.0 is required to achieve higher compressive strengths. Sathonawaphak et al. [27] stated that geopolymers produced with fly ash/alkaline activator ratios in the range of 1.4–2.3 displayed compressive strengths, ranging from 42 to 52 MPa. Their study noted that the optimum Na_2_SiO_3_/NaOH ratio was 1.5. Rattanasak et al. [28] concluded that the use of a Na_2_SiO_3_/NaOH ratio of 1.0 produced a product with a compressive strength as high as 70 MPa. However, Hardjito [29] showed that the use of a Na_2_SiO_3_/NaOH ratio of 2.5 gave the highest compressive strength, whereas a ratio of 0.4 resulted in lower compressive strength. In addition, researchers have reported that compressive strength increases as the molarity of the NaOH increases from 8 to 16 M [30]. However, Palomo et al. [25] reported that a 12 molar NaOH concentration gave higher strength than 18 M in fly ash geopolymer concrete.

Despite the past research on performance and mix design parameters of high calcium fly ash in geopolymers, there is no widely accepted procedure to determine the proportions to be mixed in concrete. Optimization by artificial intelligence tools with different algorithms [31,32] has been used for the mix design of PC concrete. The artificial neural networks (ANN) technique was used for alkali-activated concrete and found that compressive strength can be predicted with minimal error in comparison to the experimental results [33,34,35]. ANN is a statistical data modelling tool that can be trained using the available data as inputs by changing the weights with the aim to model a complex relationship between the inputs and the target outcome [36,37]. Lahoti et al. [34] investigated the effect of four influential ratios (Si/Al molar, water/solid, Al/Na molar H_2_O/Na_2_O molar) using ANN to predict the compressive strength of alkali-activated metakaolin concrete. In another study [35], ANN models with different numbers of neurons in hidden layers were investigated and predicted the compressive range of strength of alkali-activated concretes based on curing time, CaO content, NaOH concentration, and H_2_O/Na_2_O molar ratio. Researchers have been attempting to optimise the layers by using different functions for the hidden and output layers [38]. Ling et al. [33] showed a strong correlation between the ANN model predictions and the experimental results for compressive strength and setting time of high calcium fly ash geopolymer concrete.

In this study, ANN was used with three algorithms and different numbers of neurons in the hidden layer for the prediction of compressive strength of high calcium fly ash geopolymer concrete based on the data obtained from the literature. Having assessed the available mix design parameters, the water/solid ratio, alkaline activator/fly ash ratio, Na_2_SiO_3_/NaOH ratio and NaOH molarity were identified as the most influential parameters for compressive strength prediction. Although the curing temperature was reviewed and analysed for the database, the strength development over time did not identify it as an influential parameter. Based on the parameters identified, a novel standard process to find the mix proportions for a high calcium fly ash geopolymer concrete was developed, and the effectiveness of achieving a specified compressive strength was tested and validated through laboratory experiments.

## 2. Significance of Research

Although fly ash geopolymer concrete has been used in structural members and commercialised as a construction material, the mix design process is still unclear because of the many variables involved. Almost all the proposed methods employ different techniques specific for the particular situation and cannot be used as a standard method. The missing link identified is that there is no unique mix design guideline for high calcium fly ash geopolymer concrete. This research addresses the identified gap and proposes a standard mix design procedure using a machine learning technique. The validation of the technique demonstrates that the novel method developed can be used with confidence to calculate mix proportions for compressive strength in the range of 25–45 MPa.

## 3. Geopolymer Concrete Database

A database was established using the published research/literature up to 2019 (inclusive) on high calcium fly ash geopolymer concrete scrutinising it for compressive strength at 28 days. The database included only 100% high calcium fly ash concrete mixes and did not consider mortar or phase mixes, and excluded the blended high calcium fly ash composites. This selection criteria was adopted to develop a mix design procedure that could predict the standard 28-day compressive strength for high calcium fly ash geopolymer concrete more accurately. The present database consists of compressive strength values obtained from 166 concrete mix designs, Table 1.

The kurtosis values in Table 2 indicate that all the variables did not have very narrow distributions with most of the data points in the centre. When the kurtosis value was less than (−1), it showed a too flat distribution (i.e., Na_2_SiO_3_/NaOH ratio for this study) [50]. Skewness for all parameters ranged from (−0.557) to 0.521, indicating symmetrical data points with respect to the extent to which the variables’ distribution was symmetrical.

### Artificial Neural Network Model

ANN has three main layers, namely input layer, hidden layer and output layer with weights [33]. The inputs are the influential parameters collected in the database (Table 1) while “weights” give an indication of the relationship of inputs and the outputs. Equations (1) and (2) [29] are used to calculate weighted sums of inputs and outputs, respectively.

(1) $${\left(net\right)}_{j}=\overset{n}{\underset{i=1}{\sum }}w_{ij}x_{i}+b\;\;\;\;$$

(2) $${\left(out\right)}_{j}=f{\left(net\right)}_{j}=\frac{1}{1+e^{-\alpha {\left(net\right)}_{j}}}$$

where (*net*)*_j_* is the weighted sum of the *j*th neuron for the input received from the preceding layer with *n* neurons, *w_ij_* is the weight between the *j*th neuron in the preceding layer, *x_i_* is the output of the *i*th neuron in the preceding layer, *b* is a fixed value as internal addition, α is a constant used to control the slope of the semilinear region.

The ANN toolbox in MATLAB was used with three different algorithms, namely the Bayesian regularization algorithm, Levenberg-Marquardt algorithm and scaled conjugate gradient algorithm to predict the compressive strength of high calcium fly ash geopolymer concrete. The developed database was divided into two subsets as training subset and testing subset. It was noted that 63−80% of data was used for a training subset while the remainder was used for the testing subset [51,52,53]. This study randomly selected 70% of data points from the database (Table 1) for the training subset and the remainder was allocated for the testing subset. During the predata processing stage, input and output variables were generalised with respect to minimum and maximum values in order to get a range between 0 and 1.

**Figure 1:**
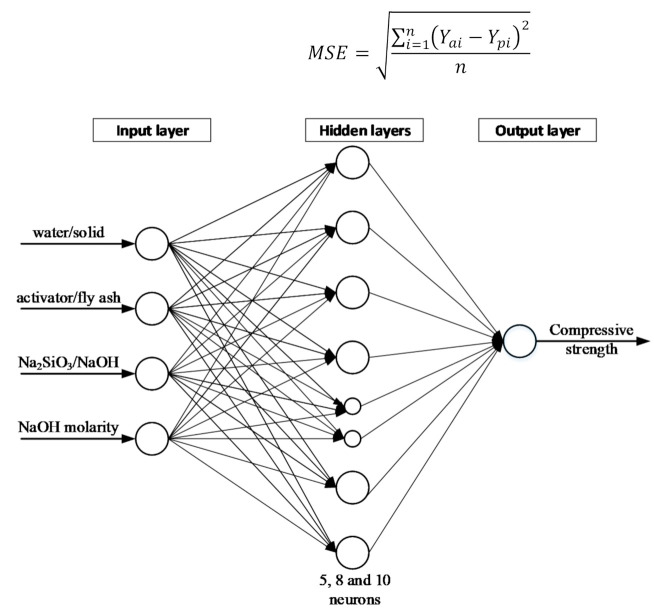
Schematic diagram of artificial neural network (ANN) model.

Figure 1 shows the ANN model construction: the 4 input variables as 4 influential parameters; the 5, 8 and 10 neurons were selected for the hidden layer; and the compressive strength was selected as the only target output. Having developed the ANN model and trained it using the training data set, next step was to evaluate the model using the testing data set. The coefficient of correlation (R) and mean square error (MSE), Equations (3) and (4) [50] were used as performance parameters in this study. In these equations, “Y” represents the compressive strength at 28 days, the “a” and “p” denote the actual and the predicted, a bar above the letter shows the mean value, and sample size if given by n.

(3) $$R=\frac{\sum_{i=1}^{n}\left(Y_{ai}-\bar{Y_{a}}\right)\left(Y_{pi}-\bar{Y_{p}}\right)}{\sqrt{\sum_{i=1}^{n}{\left(Y_{ai}-\bar{Y_{a}}\right)}^{2}}\sqrt{\sum_{i=1}^{n}{\left(Y_{pi}-\bar{Y_{p}}\right)}^{2}}}\;\;\;\;$$

(4) $$MSE=\sqrt{\frac{\sum_{i=1}^{n}{\left(Y_{ai}-Y_{pi}\right)}^{2}}{n}}\;$$

**Figure 2:**
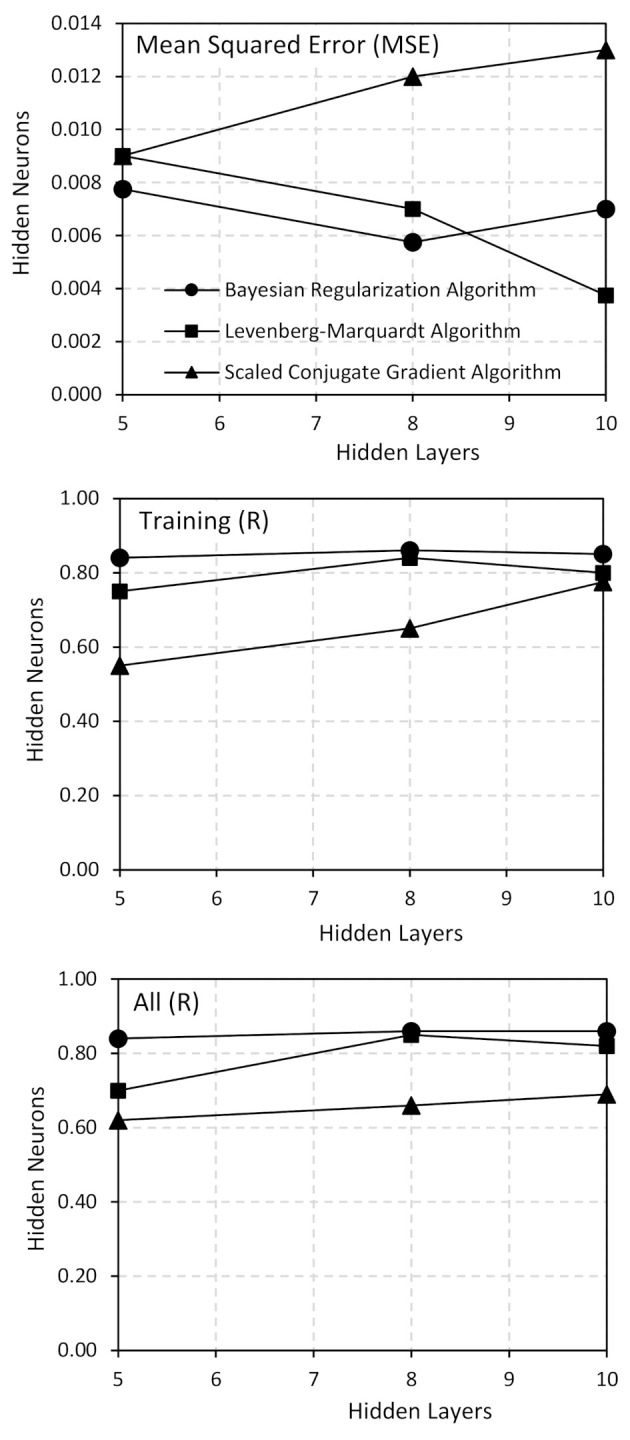
Model performance vs. number of hidden neurons for each algorithm.

Figure 2 shows the comparison of the model performance with a number of hidden neurons for different algorithms. Results showed that 8 hidden neurons along with the Bayesian regularization algorithm yielded the best correlation in the given dataset. This gave the highest R value for training, highest R value for all and second lowest value closest to zero for mean squared error.

**Figure 3:**
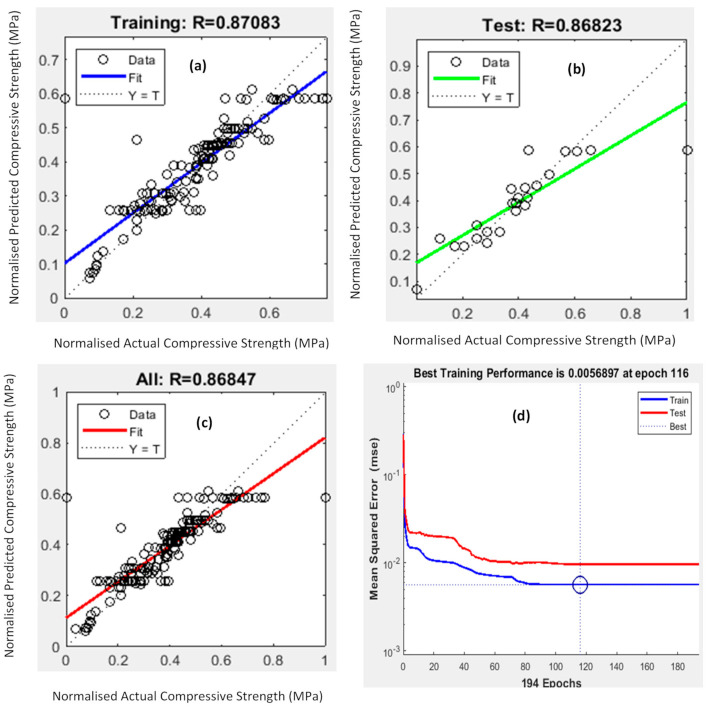
Performance of the artificial neural network (ANN) model based on (**a**) the training dataset; (**b**) test dataset; (**c**) all datasets; and (**d**) mean squared error for 8 hidden neurons and the Bayesian Regularization training algorithm.

The model performance with 8 hidden neurons along with the Bayesian Regularization algorithm is shown in Figure 3. When data points sit on the dotted straight line, it confirms the exact correlation between the predicted and compressive strength from experimental results, which is the desired outcome. It was observed that the training dataset had the best correlation with the normalised actual compressive strength when compared to the test and all plots. In addition, all three scatter plots yielded R values greater than 0.8, which meant a close relationship existed between the key mix design parameters and the 28-day compressive strength. The ANN model with the selected neurons and the algorithm had a MSE value of 0.0056897, closest to zero, confirming there was almost no error present in this predictive model.

## 4. Geopolymer Concrete Design

### 4.1. Contour Plots

**Figure 4:**
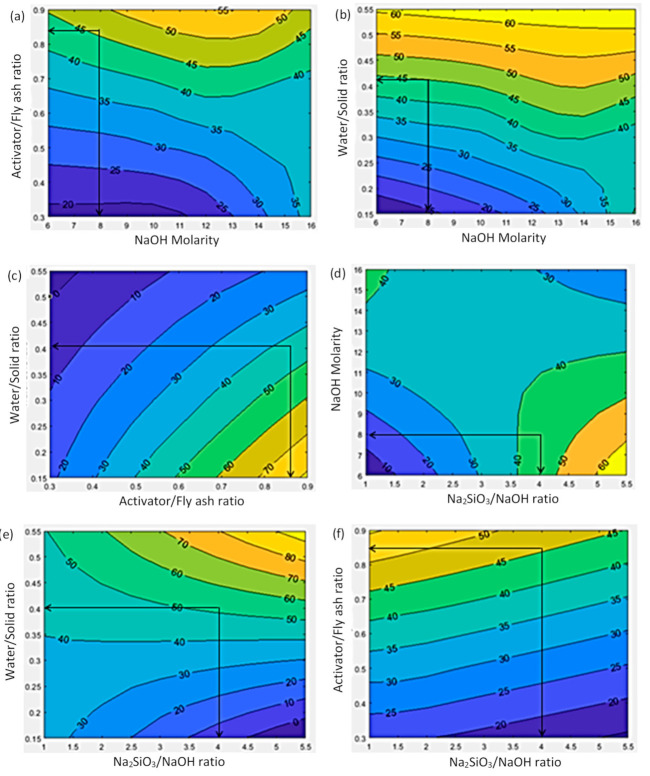
Effect of input parameters on the compressive strength (Note: for instance, the selected input parameters of 45 MPa mix is illustrated). Input parameters: (**a**) activator/fly ash ratio and NaOH molarity; (**b**) water/solid ratio and NaOH molarity; (**c**) water/solid ratio and activator/fly ash ratio; (**d**) NaOH molarity and Na_2_SiO_3_/NaOH ratio; (**e**) water/solid ratio and Na_2_SiO_3_/NaOH ratio; (**f**) activator/fly ash ratio and Na_2_SiO_3_/NaOH ratio.

The contour plots shown in Figure 4 demonstrate the intercorrelation of the selected four input variables (water/solid ratio, activator/fly ash ratio, Na_2_SiO_3_/NaOH ratio and NaOH molarity) with the target output of compressive strength. Hence, these contour maps can be used to design mix proportions for a target 28-day compressive strength for high calcium fly ash geopolymer concrete.

Water/solid ratio—Figure 4b,e depicts that there was an increasing trend for compressive strength with increased water/solid ratio. On the other hand, Figure 4c shows that even with a lower water/solid ratio a higher compressive strength could be achieved using a higher activator/fly ash ratio.

Activator/fly ash ratio—Figure 4f depicts that compressive strength had an increasing trend with an increased activator/fly ash ratio. It was possible to obtain a 20−35 MPa concrete using an activator/fly ash ratio between 0.3 to 0.6, Figure 4a. For an activator/fly ash ratio of 0.7 to 0.9 together with any NaOH molarity, 35–50 MPa compressive strength could be achieved.

Na_2_SiO_3_/NaOH ratio—Figure 4f shows that when the Na_2_SiO_3_/NaOH ratio was decreased, the compressive strength was increasing faster due to the increment in the activator/fly ash ratio. Further, by increasing the Na_2_SiO_3_/NaOH ratio with decreasing NaOH molarity, higher compressive strength could be achieved, Figure 4d.

NaOH molarity—Figure 4a illustrates that for an activator/fly ash ratio less than 0.7, compressive strength could only achieve a maximum 40 MPa irrespective of the NaOH molarity. Hence, in order to achieve higher strength, the activator/fly ash ratio needs to be in the range 0.7 to 0.9 in combination with adjustment of the NaOH molarity. Figure 4b shows a similar approach, as water/solid ratio had to be increased in combination with NaOH molarity in order to achieve higher compressive strengths.

### 4.2. Mix Design Calculation

For model validation, four high calcium fly ash geopolymer concrete mixes were designed with the targeted compressive strengths of 25, 30, 40 and 45 MPa at 28 days. A detailed calculation procedure for 45 MPa concrete mix is illustrated below. The calculated mix design variables obtained from the contour plots of Figure 4 were water/solid ratio = 0.4, activator/fly ash ratio = 0.85, Na_2_SiO_3_/NaOH ratio = 3.7 and NaOH molarity = 10 M. The 460 kg fly ash was used which is the median of database, Table 1.

(a) Alkaline activator content:
  (5) $$\frac{Activator}{Fly\;ash}=0.85\;;\;\;\frac{{\mathrm{Na}}_{2}{\mathrm{SiO}}_{3}+\mathrm{NaOH}}{Fly\;ash}=\frac{{\mathrm{Na}}_{2}{\mathrm{SiO}}_{3}}{\mathrm{NaOH}}=\;3.7\;$$
  After solving: Na_2_SiO_3_ = 307.8 kg; NaOH = 83.2 kg.

(b) Added water content:
  (6) $$\frac{Water}{Solid}=\;\frac{{\mathrm{Na}}_{2}{\mathrm{SiO}}_{3}{}_{water}+{\mathrm{NaOH}}_{water}+\mathrm{Added}\;\mathrm{Water}}{Fly\;ash+\;{\mathrm{Na}}_{2}{\mathrm{SiO}}_{3}{}_{solid}+{\mathrm{NaOH}}_{solid}}=0.4\;\;\;\;$$
  After solving: Added water (w) = 2.82 kg (Table 3).

(c) Aggregate content:
  (7) $$V_{Fly\;ash}+V_{Na_{2}SiO_{3}}+V_{NaOH}+V_{Added\;Water}+V_{Sand}+V_{Aggregate}=1$$
  (8) $$\frac{M_{Fly\;ash}}{\rho_{Fly\;ash}}+\frac{M_{Na_{2}SiO_{3}}}{\rho_{Na_{2}SiO_{3}}}+\frac{M_{NaOH}}{\rho_{NaOH}}+\frac{M_{Added\;Water}}{\rho_{Added\;Water}}+\frac{M_{Sand}}{\rho_{Sand}}+\frac{M_{Aggregate}}{\rho_{Aggregate}}=1$$
  (9) $$\frac{V_{Aggregate}}{V_{Sand}+V_{Aggregate}}=0.65$$
  After solving: $M_{Sand}=$ 467 kg and $M_{Aggregate}=$ 919.3 kg.

Similarly, Figure 4 was used to obtain the mix proportions for 25 MPa, 30 MPa and 40 MPa concretes and these were tabulated in Table 4.

### 4.3. Experimental Procedure

The high calcium fly ash obtained from an Indonesian coal power station was used for this study. The chemical composition of the fly ash, Table 5, was determined using Bruker Axs S4 Pioneer X-ray fluorescence equipment. The particle size distribution was determined using a Malvern Mastersizer analyser and the crystalline composition with a Bruker Axs D8 ADVANCE Wide Angle X-ray diffraction (XRD) instrument. The XRD analysis was performed at 40 kV, Cu Kα = 1.54178 Å wavelength, and a scanning range of 2 theta in 5–95°. Sample holders were filled using the front-loading procedure. The data obtained from XRD were interpreted using Bruker-DIFFRAC.EVA software and Rietveld analysis [54,55]. The surface area was determined using the Brunauer-Emmett-Teller method by N_2_ absorption. The crystalline and amorphous content, specific surface area, and particle size distribution are shown in Table 6.

Commercially available sodium hydroxide solution (8–10 M) and sodium silicate solution (Na_2_O = 14.7% and SiO_2_ = 29.4% by mass, specific gravity = 1.53) were used as alkaline activator in the geopolymer production. The fine aggregate and coarse aggregate were prepared with respect to the Australian Standards, AS 1141.5 [56]. River sand in an uncrushed form (specific gravity = 2.5 and fineness modulus = 2.8) was used as fine aggregate, and 10 mm grain size crushed granite aggregate (specific gravity = 2.65 and water absorption = 0.74%) was used as coarse aggregate in concrete. Demineralised water was used throughout in the mixing.

A 60 L concrete mixer was used to prepare all concrete specimens. Firstly, fly ash, sand and coarse aggregates were mixed for 4 min followed by the addition of alkaline activator and water with further mixing for 8 min. This provided a well-combined, nonsegregated concrete mix. The concrete was poured into standard cylindrical moulds (100 mm diameter × 200 mm height), then compacted using a vibration table for 1 min to remove air bubbles. All prepared concrete cylinders were kept in the laboratory under ambient conditions (23 °C temperature and 70% relative humidity) for 24 h. Afterwards, all concrete specimens were heat cured at 60 °C temperature for one day. After demoulding, all specimens were clearly labelled and stored in laboratory conditions (23 °C temperature and 70% relative humidity) until the 28 day testing. Compressive strength testing was undertaken in accordance with the ASTM C109/C109M standard using a Technotest concrete testing machine [57]. A total of 4 specimens were tested at each interval at a loading rate of 0.34 MPa/S until failure.

## 5. Experimental Results and Model Validation

The experimental results, noted in Table 7, demonstrated that the four high calcium fly ash geopolymer concrete mixes achieved close to their relevant target compressive strengths at 28 days. The M25 and M30 concrete mixes slightly exceeded the target strength while both the M40 and M45 concrete mixes displayed a slightly lower value than the expected compressive strength. Although all the mixes showed increased compressive strength from 7 to 28 days, the percentage increment was slightly different. The M25 and M30 geopolymer mixes obtained the highest strength development (~70%) while the other two concrete mixes gained ~60% strength during this period. Overall, experimental observations were in good agreement with the predicted and actual compressive strength for high calcium fly ash geopolymer concrete indicating the reliability of the mix design procedure described in this paper.

### Relationship between Mechanical Properties

The high calcium geopolymer concrete experimental data available in Table 1 was used in a regression analysis to explore the trends and correlations between elastic modulus and tensile strength with compressive strength. Residual and refined R^2^ values for selected regression models were used together with the least square method to obtain the linear regression lines to match the experimental data. Each best fit line is linked with the confidence and prediction interval bands. The prediction interval is concentrated on the specific data point while prediction lines are the focus of the confidence interval. There is a 95% chance that the actual regression line will be in the confidence interval band calculated using Equation (5) [58]. Hence, there is a 95% chance that the actual value (Y) corresponding to a particular value (X_0_,) is located within this interval, Equation (6) [58].

(10) $$Y_{pred.}\pm t_{0.05}\sqrt{\frac{\sum {\left(Y-Y_{pred.}\right)}^{2}}{n-2}}\;.\;\sqrt{\frac{1}{n}+\frac{{\left(X-\bar{X}\right)}^{2}}{SS_{x}}}$$

(11) $$Y_{pred.}\pm t_{0.05}\left(1+\sqrt{\frac{\sum {\left(Y-Y_{pred.}\right)}^{2}}{n-2}}\;\right)\;.\;\sqrt{1+\frac{1}{n}+\frac{{\left(X-\bar{X\;}\right)}^{2}}{SS_{x}}}$$

**Figure 5:**
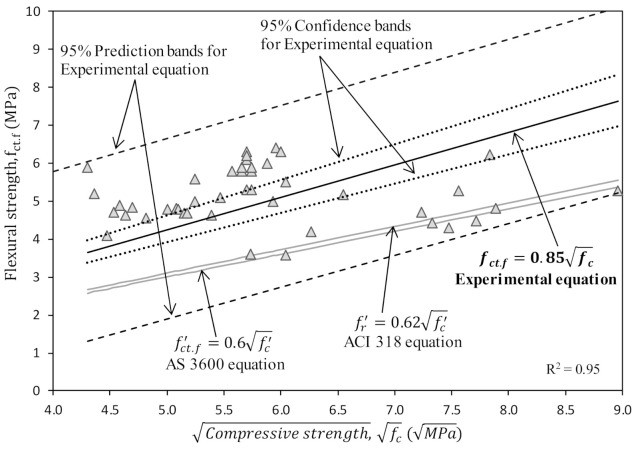
Correlation between compressive strength vs. flexural strength.

The Y_pred._ is the predicted Y values, t_0.05_ is the *t* critical value for 95% interval, n is the sample size, X is the true value while $\bar{X}$ is the mean of sample and SS_x_ is the sum of the squares of standard error of X values. The proposed regression model for the relationship between compressive and flexural strength is shown in Figure 5. Relevant equations are available in the Standards [59,60] for Portland cement concrete to evaluate the flexural strength which are used in deflection calculations. However, these equations do not lie in the 95% prediction interval bands of the regression model for experimental results. The AS 3600 [59] and ACI 318 [60] equations for flexural strength are on the lower side of the confidence interval of the proposed regression model, illustrating that the design equations of both standards underestimate the flexural strength for high calcium fly ash geopolymer concrete. Hence, the use of the current standard/code for PC concrete will achieve a conservative design for flexural members made with high calcium fly ash based geopolymer concrete.

**Figure 6:**
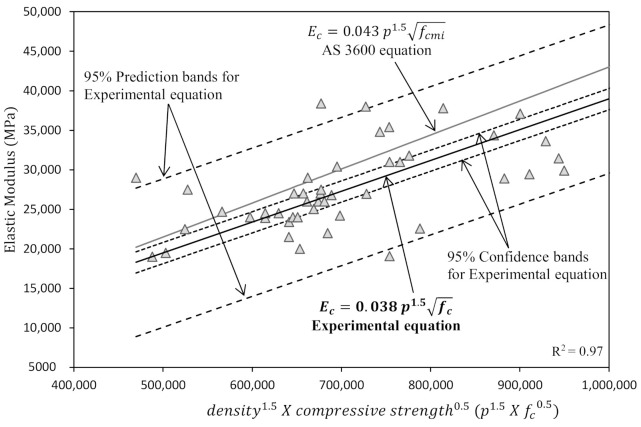
Correlation between compressive strength vs. elastic modulus.

A linear regression line with prediction and confidence interval bands to demonstrate the relationship between compressive strength and elastic modulus is shown in Figure 6. As the R^2^ value of the regression model was 0.97, this indicates that a more accurate modulus of elasticity could be achieved if the density was also considered in the equation. AS 3600 [59] and ACI 318 [60] also provide a similar design equation with the inclusion of density. Contrary to the flexural strength, the AS 3600 equation for elastic modulus lies above the upper confidence interval of proposed regression model. This implies that the available equations for PC concrete overestimate the elastic modulus of high calcium fly ash geopolymer concrete which leads to an underestimation of serviceability performance.

## 6. Summary and Conclusions

1. The algorithm for the predictive model for high calcium fly ash geopolymer concrete mix design was developed using artificial neural networks in order to determine the relationship between the four key parameters identified, namely water/solid ratio, alkaline activator/binder ratio, Na_2_SiO_3_/NaOH ratio and NaOH molarity, and the 28-day compressive strength of geopolymer concrete.
2. A new standard mix design procedure was developed for high calcium fly ash geopolymer concrete using contour plots generated in the MATLAB programming environment, and demonstrated through detailed calculation to ascertain the mix proportions for 45 MPa target compressive strength at 28 days.
3. Good correlation between the experimental results and the compressive strengths calculated from contour plots validated the developed novel mix design method for high calcium fly ash geopolymer concrete. Thus, the proposed method is suitable for calculating mix proportions with confidence for a target compressive strength at 28 days in the range of 25–45 MPa.
4. A statistical regression model was developed using the database to provide new design equations to predict tensile strength and elastic modulus of high calcium fly ash geopolymer concrete based on the 28-day compressive strengths obtained.
5. The design equations available in AS 3600 and ACI 318 standards for Portland cement concrete provide a conservative design for tensile strength in high calcium fly ash geopolymer concrete. However, AS 3600 design equation overestimates the elastic modulus for geopolymer concrete.
6. The present study suggests preliminary amendments to the available design standards for Portland cement concrete to design high calcium fly ash geopolymer concrete structural elements with better serviceability performance. However, further investigations are required prior to implementing them in the standards/codes.

## Figures and Tables

**Table 1:** Mix design database for high calcium fly ash geopolymer concrete.

Fly Ash (kg)	Aggregate (kg)		Activator (kg)		Added Water (kg)	Solid % in Na_2_SiO_3_		NaOH Molarity	Heat Curing [Ambient Curing]		Comp. Strength (MPa)	Flexural Strength (MPa)	Elastic Modulus (GPa)	Ref.
	Coarse	Fine	NaOH	Na_2_SiO_3_		SiO_2_	Na_2_O		Time	°C				
414	1091	588	104	104	0	32.9	15.3	10 M	24 h	60	46.67	–	31.00	[16]
414	1091	588	104	104	0	32.9	15.3	15 M	24 h	60	54.40	–	37.80	
414	1091	588	104	104	0	32.9	15.3	20 M	24 h	60	43.42	–	38.00	
414	1091	588	69	138	0	32.9	15.3	10 M	24 h	60	40.09	–	24.20	
414	1091	588	69	138	0	32.9	15.3	15 M	24 h	60	48.18	–	31.00	
414	1091	588	69	138	0	32.9	15.3	20 M	24 h	60	49.50	–	31.80	
414	1091	588	104	104	0	32.9	15.3	10 M	24 h	[23]	39.67	–	30.40	
414	1091	588	104	104	0	32.9	15.3	15 M	24 h	[23]	45.34	–	34.80	
414	1091	588	104	104	0	32.9	15.3	20 M	24 h	[23]	37.64	–	38.40	
414	1091	588	69	138	0	32.9	15.3	10 M	24 h	[23]	33.80	–	23.40	
414	1091	588	69	138	0	32.9	15.3	15 M	24 h	[23]	39.02	–	26.80	
414	1091	588	69	138	0	32.9	15.3	20 M	24 h	[23]	46.69	–	35.40	
523	1124	459	118	118	0	28.7	11.7	10 M	24 h	[23]	36.5	5.5	22	[39]
500	1166	475	113	113	0	28.7	11.7	10 M	24 h	[23]	33.0	5.3	26	
478	1211	490	108	108	0	28.7	11.7	10 M	24 h	[23]	26.0	4.8	24	
470	1161	474	118	118	0	28.7	11.7	10 M	24 h	[23]	32.5	6.1	24	
450	1201	489	113	113	0	28.7	11.7	10 M	24 h	[23]	32.0	5.8	24	
430	1245	504	108	108	0	28.7	11.7	10 M	24 h	[23]	27.5	5.6	23.9	
428	1191	487	118	118	0	28.7	11.7	10 M	24 h	[23]	32.0	5.9	21.5	
409	1231	501	113	113	0	28.7	11.7	10 M	24 h	[23]	29.9	5.1	24.5	
391	1273	515	108	108	0	28.7	11.7	10 M	24 h	[23]	27.5	5	24.5	
392	1216	497	118	118	0	28.7	11.7	10 M	24 h	[23]	25.0	4.8	24.7	
375	1255	511	113	113	0	28.7	11.7	10 M	24 h	[23]	21.0	4.9	27.5	
359	1296	525	108	108	0	28.7	11.7	10 M	24 h	[23]	20.0	4.1	22.5	
523	1126	460	118	118	0	28.7	11.7	15 M	24 h	[23]	35.5	6.4	27	
500	1168	475	113	113	0	28.7	11.7	15 M	24 h	[23]	32.5	6.3	27	
478	1212	491	108	108	0	28.7	11.7	15 M	24 h	[23]	31.0	5.8	20	
470	1163	475	118	118	0	28.7	11.7	15 M	24 h	[23]	36.0	6.3	26	
450	1203	490	113	113	0	28.7	11.7	15 M	24 h	[23]	34.5	6	27.5	
430	1246	505	108	108	0	28.7	11.7	15 M	24 h	[23]	33.0	5.8	26	
428	1193	487	118	118	0	28.7	11.7	15 M	24 h	[23]	32.5	6.2	27	
409	1232	502	113	113	0	28.7	11.7	15 M	24 h	[23]	33.0	5.9	29	
391	1274	516	108	108	0	28.7	11.7	15 M	24 h	[23]	32.5	5.3	25.1	
392	1218	498	118	118	0	28.7	11.7	15 M	24 h	[23]	18.5	5.9	19	
375	1257	512	113	113	0	28.7	11.7	15 M	24 h	[23]	19.0	5.2	19.5	
359	1298	525	108	108	0	28.7	11.7	15 M	24 h	[23]	16.0	5.1	29	
390	1092	585	67	167	0	30.0	9.0	8 M	28 days	[25]	23.4	–	–	[40]
390	1092	585	67	167	0	30.0	9.0	10 M	28 days	[25]	25.0	–	–	
390	1092	585	67	167	0	30.0	9.0	12 M	28 days	[25]	28.2	–	–	
390	1092	585	67	167	0	30.0	9.0	14 M	28 days	[25]	31.8	–	–	
390	1092	585	67	167	0	30.0	9.0	16 M	28 days	[25]	32.2	–	–	
390	1092	585	67	167	0	30.0	9.0	18 M	28 days	[25]	30.3	–	–	
300	1684	681	51.4	129	0	29.4	14.7	14 M	24 h	60	25.8	4.81	–	[41]
300	1684	681	51.4	129	0	29.4	14.7	14 M	24 h	60	23.2	4.56	–	
300	1684	681	51.4	129	0	29.4	14.7	14 M	24 h	60	21.5	4.63	–	
300	1684	681	51.4	129	0	29.4	14.7	14 M	24 h	60	26.8	4.69	–	
300	1684	681	51.4	129	0	29.4	14.7	14 M	24 h	60	20.5	4.72	–	
300	1684	681	51.4	129	0	29.4	14.7	14 M	24 h	60	22.0	4.85	–	
600	1087	572	89.1	223	0	29.4	14.7	14 M	24 h	60	26.5	4.68	–	
600	1087	572	89.1	223	0	29.4	14.7	14 M	24 h	60	29.0	4.65	–	
600	1087	572	89.1	223	0	29.4	14.7	8 M	24 h	60	27.0	–	–	
600	1087	572	89.1	223	0	29.4	14.7	8 M	24 h	60	25.0	–	–	
600	1087	572	89.1	223	0	29.4	14.7	8 M	24 h	60	22.5	–	–	
600	1087	572	89.1	223	0	29.4	14.7	8 M	24 h	60	28.5	–	–	
600	1087	572	89.1	223	0	29.4	14.7	8 M	24 h	60	22.0	–	–	
600	1087	572	89.1	223	0	29.4	14.7	8 M	24 h	60	30.0	–	–	
494	858	691	198	198	0	30.0	15.0	14 M	72 h	60	59.5	4.48	33.63	[42]
494	858	691	198	198	0	30.0	15.0	14 M	72 h	60	52.3	4.72	34.37	
494	858	691	198	198	0	30.0	15.0	14 M	72 h	60	55.9	4.3	37.10	
494	858	691	198	198	0	30.0	15.0	14 M	72 h	60	80.4	5.27	42.87	
494	858	691	198	198	0	30.0	15.0	14 M	72 h	60	61.4	6.23	31.44	
494	858	691	198	198	0	30.0	15.0	14 M	72 h	60	39.2	4.19	19.06	
494	858	691	198	198	0	30.0	15.0	14 M	72 h	60	53.7	4.43	28.91	
494	858	691	198	198	0	30.0	15.0	14 M	72 h	60	36.5	3.58	26.97	
494	858	691	198	198	0	30.0	15.0	14 M	72 h	60	57.2	5.27	29.44	
494	858	691	198	198	0	30.0	15.0	14 M	72 h	60	42.8	5.18	22.56	
494	858	691	198	198	0	30.0	15.0	14 M	72 h	60	62.2	4.83	29.89	
450	1150	500	108	162	0	30.3	12.3	12 M	48 h	60	35.2	5	–	[43]
450	1036	500	108	162	0	30.3	12.3	12 M	48 h	60	32.9	3.6	–	
550	838	600	95	239	0	30.0	12.0	8 M	24 h	60	33.2	–	–	[44]
550	838	600	95	239	0	30.0	12.0	8 M	28 day	[29]	35.6	–	–	
550	838	600	95	239	0	30.0	12.0	10 M	24 h	60	35.4	–	–	
550	838	600	95	239	0	30.0	12.0	10 M	28 day	[29]	36.7	–	–	
550	838	600	95	239	0	30.0	12.0	12 M	24 h	60	42.4	–	–	
550	838	600	95	239	0	30.0	12.0	12 M	28 day	[29]	39.7	–	–	
550	838	600	95	239	0	30.0	12.0	14 M	24 h	60	40.1	–	–	
550	838	600	95	239	0	30.0	12.0	14 M	28 day	[29]	38.7	–	–	
550	838	600	95	239	0	30.0	12.0	8 M	24 h	60	34.7	–	–	
550	838	600	95	239	0	30.0	12.0	8 M	28 day	[29]	36.2	–	–	
550	838	600	95	239	0	30.0	12.0	10 M	24 h	60	34.3	–	–	
550	838	600	95	239	0	30.0	12.0	10 M	28 day	[29]	37.1	–	–	
550	838	600	95	239	0	30.0	12.0	12 M	24 h	60	41.3	–	–	
550	838	600	95	239	0	30.0	12.0	12 M	28 day	[29]	38.9	–	–	
550	838	600	95	239	0	30.0	12.0	14 M	24 h	60	42.3	–	–	
550	838	600	95	239	0	30.0	12.0	14 M	28 day	[29]	38.5	–	–	
550	838	600	95	239	0	30.0	12.0	8 M	24 h	60	36.3	–	–	
550	838	600	95	239	0	30.0	12.0	8 M	28 day	[29]	35.3	–	–	
550	838	600	95	239	0	30.0	12.0	10 M	24 h	60	36.1	–	–	
550	838	600	95	239	0	30.0	12.0	10 M	28 day	[29]	36.3	–	–	
550	838	600	95	239	0	30.0	12.0	12 M	24 h	60	42.2	–	–	
550	838	600	95	239	0	30.0	12.0	12 M	28 day	[29]	45.3	–	–	
550	838	600	95	239	0	30.0	12.0	14 M	24 h	60	40.2	–	–	
550	838	600	95	239	0	30.0	12.0	14 M	28 day	[29]	39.6	–	–	
550	838	600	95	239	0	30.0	12.0	8 M	24 h	60	34.4	–	–	
550	838	600	95	239	0	30.0	12.0	8 M	28 day	[29]	36.3	–	–	
550	838	600	95	239	0	30.0	12.0	10 M	24 h	60	35.4	–	–	
550	838	600	95	239	0	30.0	12.0	10 M	28 day	[29]	38.3	–	–	
550	838	600	95	239	0	30.0	12.0	12 M	24 h	60	43.4	–	–	
550	838	600	95	239	0	30.0	12.0	12 M	28 day	[29]	44.3	–	–	
550	838	600	95	239	0	30.0	12.0	14 M	24 h	60	39.4	–	–	
550	838	600	95	239	0	30.0	12.0	14 M	28 day	[29]	38.3	–	–	
550	838	600	95	239	0	30.0	12.0	8 M	24 h	60	33.1	–	–	
550	838	600	95	239	0	30.0	12.0	8 M	28 day	[29]	33.5	–	–	
550	838	600	95	239	0	30.0	12.0	10 M	24 h	60	35.1	–	–	
550	838	600	95	239	0	30.0	12.0	10 M	28 day	[29]	35.5	–	–	
550	838	600	95	239	0	30.0	12.0	12 M	24 h	60	42.2	–	–	
550	838	600	95	239	0	30.0	12.0	12 M	28 day	[29]	41.5	–	–	
550	838	600	95	239	0	30.0	12.0	14 M	24 h	60	40.2	–	–	
550	838	600	95	239	0	30.0	12.0	14 M	28 day	[29]	37.5	–	–	
550	838	600	95	239	0	30.0	12.0	8 M	24 h	60	34.2	–	–	
550	838	600	95	239	0	30.0	12.0	8 M	28 day	[29]	35.5	–	–	
550	838	600	95	239	0	30.0	12.0	10 M	24 h	60	36.2	–	–	
550	838	600	95	239	0	30.0	12.0	10 M	28 day	[29]	37.5	–	–	
550	838	600	95	239	0	30.0	12.0	12 M	24 h	60	41.4	–	–	
550	838	600	95	239	0	30.0	12.0	12 M	28 day	[29]	40.5	–	–	
550	838	600	95	239	0	30.0	12.0	14 M	24 h	60	40.8	–	–	
550	838	600	95	239	0	30.0	12.0	14 M	28 day	[29]	38.4	–	–	
310	1204	649	48.6	121.5	0	34.7	16.2	10 M	24 h	80	44.4	–	–	[45]
350	1250	650	41	103	0	29.8	14.7	8 M	7 day	[28]	19.0	–	–	[46]
350	1250	650	41	103	0	29.8	14.7	8 M	7 day	[28]	26.0	–	–	
350	1250	650	41	103	0	29.8	14.7	8 M	7 day	[28]	23.5	–	–	
350	1250	650	41	103	0	29.8	14.7	8 M	7 day	[28]	22.5	–	–	
350	1250	650	41	103	0	29.8	14.7	8 M	7 day	[28]	17.8	–	–	
350	1250	650	41	103	0	29.8	14.7	8 M	7 day	[28]	21.5	–	–	
350	1250	650	41	103	0	29.8	14.7	8 M	7 day	[28]	19.0	–	–	
350	1250	650	41	103	0	29.8	14.7	8 M	7 day	[28]	13.0	–	–	
350	1250	650	41	103	0	29.8	14.7	8 M	7 day	[28]	12.0	–	–	
350	1250	650	41	103	0	29.8	14.7	8 M	24 h	60	32.5	–	–	
350	1250	650	41	103	0	29.8	14.7	8 M	24 h	60	33.5	–	–	
350	1250	650	41	103	0	29.8	14.7	8 M	24 h	60	31.0	–	–	
350	1250	650	41	103	0	29.8	14.7	8 M	24 h	60	24.7	–	–	
350	1250	650	41	103	0	29.8	14.7	8 M	24 h	60	22.0	–	–	
350	1250	650	41	103	0	29.8	14.7	8 M	24 h	60	25.0	–	–	
350	1250	650	41	103	0	29.8	14.7	8 M	24 h	60	23.5	–	–	
350	1250	650	41	103	0	29.8	14.7	8 M	24 h	60	16.0	–	–	
350	1250	650	41	103	0	29.8	14.7	8 M	24 h	60	15.0	–	–	
383	1379	567	54.5	137.00	0	32.4	13.5	12 M	7 day	[30]	20.0	–	–	[44]
527	1159	522	53.3	133.33	0	32.4	13.5	12 M	7 day	[30]	19.0	–	–	
530	1070	505	51.6	128.59	0	32.4	13.5	12 M	7 day	[30]	16.0	–	–	
450	1200	600	80	120	0	32.4	13.5	10 M	7 day	[25]	18.5	–	–	[47]
450	1200	600	80	120	0	32.4	13.5	12 M	7 day	[25]	27.0	–	–	
450	1200	600	80	120	0	32.4	13.5	14 M	7 day	[25]	29.3	–	–	
410	1143	521.8	110	120	2.3	32.4	13.5	10 M	7 day	[25]	16.2	–	–	
410	1143	521.8	110	120	2.3	32.4	13.5	12 M	7 day	[25]	25.0	–	–	
410	1143	521.8	110	120	2.3	32.4	13.5	14 M	7 day	[25]	22.5	–	–	
350	1200	645	41	103	35	29.8	14.7	8 M	3 day	[35]	19.0	–	–	[48]
350	1200	645	41	103	35	29.8	14.7	8 M	24 h	65	49.0	–	–	
350	1200	645	41	103	35	29.8	14.7	8 M	3 day	55	48.0	–	–	
500	1000	750	125	125	0	30.0	15.0	14 M	72 h	80	51.0	–	–	[49]
500	1000	750	125	125	0	30.0	15.0	14 M	72 h	80	53.0	–	–	
500	1000	750	125	125	0	30.0	15.0	14 M	72 h	80	50.0	–	–	
500	1000	750	125	125	0	30.0	15.0	14 M	72 h	80	50.0	–	–	
500	1000	750	125	125	0	30.0	15.0	14 M	72 h	80	52.0	–	–	
500	1000	750	125	125	0	30.0	15.0	14 M	72 h	80	44.0	–	–	

**Table 2:** Statistics for input and target parameters.

Variable	Minimum	Maximum	Average	SD	Skewness	Kurtosis
Water/solid	0.123	0.330	0.263	0.045	−0.557	−0.243
Activator/fly ash	0.340	0.802	0.549	0.105	0.433	0.388
Na_2_SiO_3_/NaOH	0.500	2.516	1.878	0.754	−0.488	−1.569
NaOH molarity	8	20	11.8	3.12	0.521	−0.118
Compressive strength (MPa)	12	80.4	32.8	12.4	0.218	0.779

**Table 3:** Data tabulation: calculating added water content.

	Na_2_SiO_3_	NaOH	Extra Water	Binder	Total
Solid	120.1	25.8	0	460	605.9
Water	187.7	57.4	w	0	245.1 + w

**Table 4:** Mix proportions of high calcium fly ash geopolymer concrete.

Mix Notation	Target Strength	Mix Design Variables							
		Water/Solid		Activator/Fly Ash		Na_2_SiO_3_/NaOH		NaOH Molarity	
M25	25 MPa	0.25		0.42		1.5		10 M	
M30	30 MPa	0.28		0.50		2.0		10 M	
M40	40 MPa	0.35		0.70		3.5		10 M	
M45	45 MPa	0.42		0.85		4.0		8 M	
**Mix Notation**	**Target** **Strength**	**Mix Proportions (kg/m^3^)**							
		**Fly Ash**	**Sand**		**Aggregates**	**Na_2_SiO_3_**	**NaOH**		**Added Water**
M25	25 MPa	460	500.8		985.8	115.9	77.3		93.7
M30	30 MPa	460	495.7		975.8	153.3	76.7		75.5
M40	40 MPa	460	480.3		945.5	250.5	71.5		33.1
M45	45 MPa	460	467		919.3	307.8	83.2		2.82

**Table 5:** Chemical composition of high calcium fly ash.

Source Material	Component (wt.%)											
	SiO_2_	Al_2_O_3_	Fe_2_O_3_	CaO	P_2_O_5_	TiO_2_	MgO	K_2_O	SO_3_	MnO	Na_2_O	LOI ^a^
Fly ash	38.7	20.8	5.3	26.6	0.15	0.45	1.5	2.6	2.1	0.5	1.2	0.1

^a^ Loss on ignition (unburnt carbon content).

**Table 6:** Physical and mineralogical properties of high calcium fly ash.

Properties Investigated		Fly Ash
Specific gravity		2.15
BET Surface area, (m^2^/kg)		2619
Fineness (%)	at 10 microns	45.2
	at 20 microns	64.1
	at 45 microns	85.9
Amorphous content (%)		67.1
Crystalline content (%)		32.8

**Table 7:** Measured compressive strength (MPa).

Concrete Type	7-Day	28-Day
M25	19.0 ± 0.6	27.2 ± 0.8
M30	23.1 ± 0.9	33.1 ± 1.2
M40	22.9 ± 0.7	38.1 ± 0.5
M45	32.7 ± 0.9	44.1 ± 0.8

## Data Availability

All data are included in the manuscript.
